# The Influence of Neuron-Extrinsic Factors and Aging on Injury Progression and Axonal Repair in the Central Nervous System

**DOI:** 10.3389/fcell.2020.00190

**Published:** 2020-03-25

**Authors:** Theresa C. Sutherland, Cédric G. Geoffroy

**Affiliations:** Department of Neuroscience and Experimental Therapeutics, Texas A&M Health Science Center, Bryan, TX, United States

**Keywords:** aging, neuron-extrinsic factors, inflammation, microglia, astrocytes, extra-cellular matrix, signaling, spinal cord injury

## Abstract

In the aging western population, the average age of incidence for spinal cord injury (SCI) has increased, as has the length of survival of SCI patients. This places great importance on understanding SCI in middle-aged and aging patients. Axon regeneration after injury is an area of study that has received substantial attention and made important experimental progress, however, our understanding of how aging affects this process, and any therapeutic effort to modulate repair, is incomplete. The growth and regeneration of axons is mediated by both neuron intrinsic and extrinsic factors. In this review we explore some of the key extrinsic influences on axon regeneration in the literature, focusing on inflammation and astrogliosis, other cellular responses, components of the extracellular matrix, and myelin proteins. We will describe how each element supports the contention that axonal growth after injury in the central nervous system shows an age-dependent decline, and how this may affect outcomes after a SCI.

## Introduction

Spinal Cord Injury (SCI) is the second most common cause of paralysis, following stroke, and places a significant and life-long burden on patients. Globally, the SCI incidence rate is approximately 13 cases per 100,000 population, with an average of 0.93 million new cases occurring each year (James et al., 2019). In the United States the incidence is approximately 54 cases per million people, with around 17,730 new cases each year (Singh et al., 2014; NSCISC, 2019). In the past decades, SCI has shown an important shift in the demographic population affected. There is a peak in SCI in a younger cohort (<30 years) followed by a second peak in an aging cohort (65 years and over) (Devivo, 2012; Singh et al., 2014). The incidence of SCI in middle-aged and aging populations is increasing (Devivo and Chen, 2011; Devivo, 2012; Singh et al., 2014). In the United States the average age of incidence of SCI is ∼43 (NSCISC, 2019) and the average age of people living with SCI who reported paralysis due to a SCI is ∼48 (Christopher and Dana Reeve Foundation, 2009). The likelihood of complications and co-morbidities increases with age; this is accompanied by a decline in prognosis and rehabilitation outcome (Devivo et al., 1990).

Age has been recognized as an important factor influencing the severity and outcome of spinal cord injury. Indeed, studies have demonstrated that old animals recover less than their younger counterparts after SCI (Gwak et al., 2004; Siegenthaler et al., 2008; von Leden et al., 2017), however, the exact mechanisms involved are unknown. The majority of studies in SCI, especially in the field of axon growth and regeneration, use young animals to model the injury, due to the relative ease compared to older animal models. Experimentally the average age of rat used to model SCI is 96 days old (±28) and less than 0.35% of the animals are 12 months, which corresponds to ∼40 years old in humans, or older (Nielson et al., 2014; Fouad et al., 2019). This creates a dichotomy between the experimental models and the aging human SCI population that may have significant impacts on the translation of research into clinically relevant therapeutic strategies. Therefore, a better understanding of the impacts of age and aging on SCI recovery and repair at the cellular and molecular level is imperative, especially to the continued development and tailoring of therapeutic interventions.

Axon regeneration is an important part of recovering function after injury to the nervous system. Long-distance axon regeneration and substantial functional recovery has been observed in the adult mammalian peripheral nervous system (PNS) (Huebner and Strittmatter, 2009). PNS neurons have demonstrated an ability to modulate gene expression in response to injury, in order to promote axon growth (He and Jin, 2016). In contrast, the central nervous system (CNS) shows little to no tangible regenerative potential after injury and limited plasticity. One major limiting factor for the success of regeneration in mature CNS axons is their poor intrinsic regenerative capacity (Lu et al., 2014). This is combined with complex interactions of both neuron-intrinsic and extrinsic factors in the injury that result in a failure to successfully regenerate healthy axons. Corticospinal neurons, in particular, have a very low capacity for axon regrowth, even after manipulations to create a permissive extrinsic environment, neutralization of inhibitory molecules or tissue grafts (Lu et al., 2014). In some cases, it has been observed that while CNS axons severed by trauma show immediate local sprouting, this progresses to become swollen, disorganized and dystrophic (He and Jin, 2016). The lack of intrinsic regenerative ability of CNS neurons results in the poor regeneration, and limited functional recovery observed after SCI. This lack of intrinsic capacity is also compounded by interaction with a wide range of inhibitory neuron-extrinsic factors.

Despite the rapid progress in understanding the cellular and molecular regulation of axon growth after injury, a major gap exists in our knowledge of how aging impacts CNS axon growth. An age-dependent decline in axon growth has been reported in a variety of model organisms, including *C. elegans* (Byrne et al., 2014; Hammarlund and Jin, 2014), zebrafish (Graciarena et al., 2014), and mammals PNS (Pestronk et al., 1980; Verdú et al., 1995, 2000). The minimal natural ability of CNS axons to regenerate under normal conditions makes the observation of further reduction with age extremely difficult. Only recently has this age-dependent decline in axon regeneration potential been shown after SCI (Geoffroy et al., 2016).

The relationship between age/aging and axon growth is complicated and multifactorial. Both neuron-intrinsic and extrinsic factors play significant roles in the capability for axon regeneration after damage, and the age-dependent weakening of this capability. In the following review, we examine the current evidence for an age-dependent decline in axon growth after CNS injury, with specific focus on the role of neuron-extrinsic factors. The neuron-intrinsic factors have been addressed in a previous review, and will only briefly be discussed (Geoffroy et al., 2017). We will discuss how inflammation, astrogliosis, other cells around the injury site, the components of the extracellular matrix and the myelin proteins are altered with age and SCI, and their respective potential involvement in the age-dependent axon regeneration decline. Understanding the underlying mechanisms of age-dependent decline in recovery potential is critical for the development of therapies to stimulate repair in patients regardless of age.

### Evidence for Age Dependent Axon Growth Decline

There is growing evidence for an age-dependent decline in axon growth, and regeneration potential, across a variety of model organisms. In aging zebrafish, axon regeneration has been shown to occur at a reduced speed and with increased latency (Graciarena et al., 2014). A similar decline in axon regeneration efficiency has been observed in *C. elegans* (Zou et al., 2013; Hammarlund and Jin, 2014) with both models putatively linked to altered neuron-intrinsic mechanisms.

In mammalian models, regrowth of aged peripheral axons is delayed, slower and less effective than that in younger animals (Verdú et al., 1995; Kerezoudi and Thomas, 1999; Kang and Lichtman, 2013). Pharmaceutical denervation also failed to elicit any growth response in aged (28 month old) rats (Pestronk et al., 1980). While the exact mechanisms and etiology of the decline of PNS regeneration with are unclear (Willcox and Scott, 2004), both neuron-intrinsic or extrinsic mechanisms seem to be at play. The processes of myelin clearance is also delayed in aging and is associated with decreases in fibers in the affected nerves (Vaughan, 1992; Kang and Lichtman, 2013). Adult DRG neurons *in vitro* present approximately 30% slower growth than their neonate counterparts (Lamoureux et al., 2010). The axonal atrophy observed in aged nerve fibers may be attributable to the reduced expression and transport of cytoskeletal proteins (Verdú et al., 2000), reduction in the rate of axonal transport (Stromska and Ochs, 1982; Kerezoudi and Thomas, 1999) as well as the decreased expression of nerve growth factor receptors (Parhad et al., 1995). Peripheral neuropathies resulting from these axonal changes with age are common in elderly populations (Cho et al., 2006). The age-related changes and decline are ambiguous, and do not progress linearly with age, exhibiting variation between studies (Verdú et al., 2000).

The relationship between age and axon regeneration in the CNS has received much less attention due to its already limited natural ability of CNS axons to regenerate. There is growing evidence for the same age-dependent decline that is seen in the PNS. Developmental studies have shown that changes in both the neuron-extrinsic environment of the spinal cord and intrinsic changes can reduce regeneration with age (Blackmore and Letourneau, 2006). In mammalian models of SCI, aging reduces locomotor recovery (Gwak et al., 2004) and is linked to changes in inflammation (von Leden et al., 2017) and myelination (Siegenthaler et al., 2008). Additionally, aging has varied effects on axon growth depending on the axon tract examined, with reduced rostral sprouting in the majority of major tracts at the lesion site (Jaerve et al., 2011). The neuronal deletion of phosphatase and tensin homolog (PTEN), a negative regulator of mammalian target of rapamycin (mTOR), has emerged as an effective target to promote the regeneration of the cortical spinal tract (CST) axons after an injury in young animals (Sun et al., 2011; Geoffroy et al., 2015). Recently, an age-dependent decline in mammalian CNS axon regeneration has been documented using PTEN-deletion strategies (Du et al., 2015; Geoffroy et al., 2016).

The regeneration, repair and regrowth of damaged axons is a complex process that relies on both internal mechanisms and responses to external signals. A balance of intrinsic and extrinsic cues determines the innate ability of injured CNS neurons to promote axon growth, and to elongate over distance. Both neuron-intrinsic and extrinsic mechanisms are altered in the normal aging process, resulting in a decline in the growth and regenerative potential of axons. There are a myriad of neuronal-intrinsic factors that interact and can impact axon growth after damage, including different pathways (PTEN/mTOR pathway, Park et al., 2008; Sun et al., 2011; Geoffroy et al., 2015), Suppressor of Cytokine Signaling 3 (SOCS3)/Signal Transducer and Activator of Transcription 3 (STAT3), Wnt/Ryk, insulin-like growth factor (IGF)-1, tumor suppressor p53, and Krüppel-like factors (KLFs), mitochondrial function, neuronal viscosity and axonal transport. These have been addressed in depth in our previous review focusing on the changes of neuron-intrinsic mechanisms with age (Geoffroy et al., 2017). The current review will only concentrate on the neuron-extrinsic factors influencing axon regeneration.

### The Impact of Extra-Neuronal Components on Axon Growth Potential

It is widely accepted that the pathophysiology of SCI consists of two distinct components (Kwon et al., 2004; Donnelly and Popovich, 2008). The primary injury results in the disruption of axons, the surrounding glial cells and blood vessels. The secondary injury is delayed and manifests in a broad spectrum of pathologies that exacerbate the injury and can continue for years after the primary trauma (Profyris et al., 2004; Rowland et al., 2008).

**FIGURE 1:**
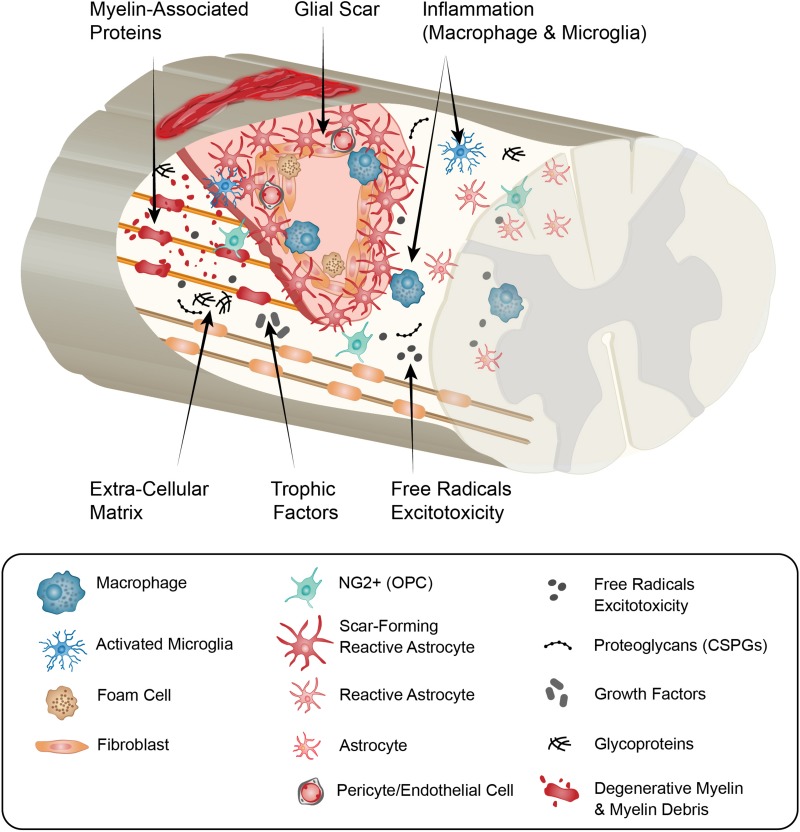
Summary schematic of the cellular and extracellular cellular factors that can influence axon growth in the chronic spinal cord injury scar and that may be altered with age. In response to traumatic spinal injury, many cellular and molecular mechanisms are activated, including an inflammatory response (neutrophils, monocytes, microglia, macrophages), astrocytic response (forming the astroglial scar), and fibroblast response (from different origins, forming the fibrotic scar). With age, the initial injury response is altered and a reduction of locomotor functional recovery is observed. There is more BSCB permeability (resulting in more blood cells and molecules entering the injury), more pro-inflammatory response (more “M1-phenotype”), reduction in wound healing (linked to the alteration in the astrocytic response), induction of cell death (including neurons and oligodendrocytes). In the more chronic phase of the injury, age keeps changing the cellular response compared to young animals (inflammatory cells, astrocytes, fibroblasts, OPCs, endothelial, pericytes) which alters the environment: there is more debris present (including axon and myelin debris), alteration in the growth factors produced, changes in ECM composition (changes in glycoproteins and proteoglycans production), and less revascularization. All of these can participate in the age-dependent decline in axon regeneration. BSCB, blood spinal cord barrier; ECM, extracellular matrix; OPCs, oligodendrocytes precursor cells.

**FIGURE 2:**
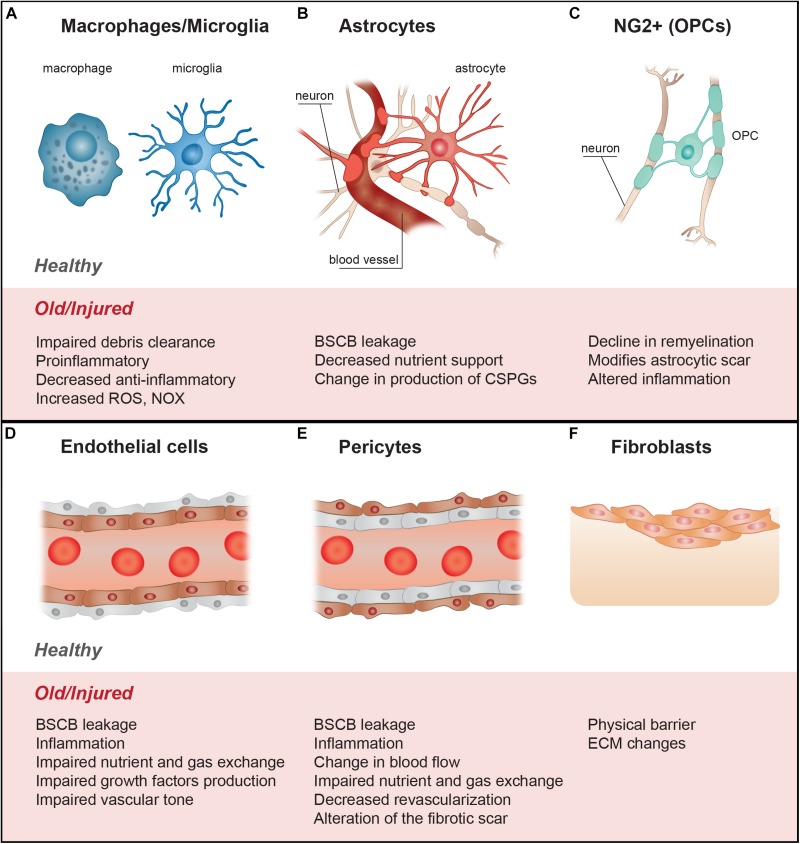
The cellular contributors to spinal cord injury altered in aging further reduce injury repair and axon growth. **(A)** Inflammatory response by microphages and microglia results in more a pro-inflammatory and less anti-inflammatory profile, associated with a reduction in debris clearance efficiency, and increase in ROS and NOX. **(B)** Astrocytes form the astroglial scar and their alteration with age and injury may include changes in ECM molecule expression profiles (growth factors, glycoproteins, proteoglycan production - CSPGs). Astrocytes become pro-inflammatory, resulting in increases in ROS and NOX. Changes in astrocyte functions also include increases in BSBC leakiness and reduction in nutrient availability. **(C)** Fibroblasts, from different origins, form the fibrotic scar; with age and injury, the profile of the ECM molecules they produce changes, making the injury more anti-regenerative, altering the injury stiffness, and making a physical barrier to growth. **(D)** Changes in the state of the endothelial cells with age can lead to increases in BSBC leakiness, infiltration of inflammatory molecules, reduction in growth factor production, and reduction in control of the vascular tone, leading to local hypoxia and reduction in nutrients entry. **(E)** Similar to endothelial cells, alteration in the functions of pericytes with age and injury may lead to vascular dysfunction, reduction in trophic support, increase in local hypoxia and decrease in angiogenesis. The roles of pericytes in the fibrotic scar and production of ECM molecules may also be altered. **(F)** With age, the proliferation and differentiation potential of OPCs is reduced, which can diminish the potential for remyelination, alter neuroinflammation and reduce neuron survival after SCI. These changes can also alter the astrocytic scar, making it more inhibitory. BSCB, blood spinal cord barrier; CSPGs, chondroitin sulfate proteoglycans; ECM, extracellular matrix; OPCs, oligodendrocytes precursor cells; NOX, NADPH-oxidase; ROS, reactive oxygen species.

A range of cell signals and processes, which are intrinsically linked, influence the lesion microenvironment and the ongoing injury response (Figure 1). These include- microvascular disruption, hemorrhage, and disruption of cellular membranes resulting in the release of cytotoxic molecules, and excess ions and glutamate which causes damage to the surrounding cells by excitotoxicity (Brown et al., 2005), oxidative stress (Potts et al., 2006), lipid peroxidation of membranes (Shetye et al., 2014), disruption of intercellular ion balance (Potts et al., 2006), and induction of cell death signals. A hallmark of the secondary injury phase is a strong and persistent pro-inflammatory cascade, characterized by neutrophil and macrophage infiltration, microglial activation and astrogliosis (Profyris et al., 2004; Potts et al., 2006; Rowland et al., 2008). The injury environment is also influenced by contributions from fibroblasts, endothelial cells, pericytes and oligodendrocyte progenitor cells (OPCs) (Figure 2). This is characterized by degenerating myelin, increases in neuro-inhibitory molecules and oxidative stressors, alterations in the extracellular matrix (ECM), and fibrotic scar formation in the affected area.

## Neuroinflammation and Phagocytes in the Central Nervous System

### Inflammation and the Immune Response in the Central Nervous System

In the inflammatory cascade occurring post-injury in the CNS, neutrophils are recruited, and endogenous microglia and astrocytes are activated. Blood monocytes migrate to the injury site and differentiate into macrophages nearly indistinguishable from activated microglia. There is a prolonged and dysregulated inflammatory response propagated by pro-inflammatory macrophages (Gensel and Zhang, 2015). Many elements of the inflammatory response have both neuroprotective and neurotoxic potential (Kwon et al., 2004). Infiltrating neutrophils produce oxidative and proteolytic enzymes that prepare the area for repair; however, the overwhelming numbers drawn to the lesion can be detrimental to the surrounding tissues. Macrophages and microglia are capable of producing factors that may help promote axonal growth, as well as molecules that are neurotoxic (Fleming et al., 2006). For in depth reviews see Hohlfeld et al. (2007).

#### Inter- and Intra-Cellular Immune Signaling in the Injured Nervous System

After neurotrauma cytokines are secreted from a variety cells, and a range of receptors are expressed on the surface of multiple cell types (Pineau and Lacroix, 2007). Microglia monitor the extracellular environment, and express a large range of cytokine and chemokine receptors (Dheen et al., 2007). Astrocytes have an active involvement in the inflammatory response, expressing receptors to a variety of signals (Sofroniew, 2015). OPCs have also been shown to be capable of modulating and influencing the immune response in EAE, a multiple sclerosis model (Falcão et al., 2018). Oligodendrocytes also express some cytokine receptors, differing from the expression profiles of OPCs, astrocytes and microglia (Sawada et al., 1993). Neural stem/progenitor cells (NSPCs) have recently been discovered to be modulators of the inflammatory response, expressing a range of receptors for cytokines and growth factors (Ziv et al., 2006). Even mature neurons can express low levels of some cytokine receptors (Sawada et al., 1993).

Mitogen activated protein kinases (MAPKs) are known to mediate many fundamental cellular processes and responses to extrinsic stressors. p38 and c-Jun N-terminal kinase (JNK) signaling cascades have been implicated in the regulation of inflammatory mediator transcription and translation, making them attractive targets in multiple disease models (Kaminska, 2005). Interestingly, both the p38 and JNK MAPK pathways are required to promote axon regeneration, and in the absence of either pathway axon regeneration will fail (Nix et al., 2011). In cerebral ischemia sustained activation of p38 has been observed in microglia and the use of a MAPK inhibitor has been found to reduce the size of the resulting infarct (Sugino et al., 2000). Similar results, and decreases in neurological deficits, have been seen in models of stroke (Barone et al., 2001). An inhibition of MAPK signaling, and resulting reduction in inflammation, has also been shown to be neuroprotective after SCI in rats (Qu et al., 2012). The mTOR pathway is also well known for its contribution to multiple cellular functions, including cell growth and metabolism, which has made it an attractive target. In the CNS, mTOR is involved in neuronal cell growth and survival, axonal and dendritic development, and synaptic plasticity (Lin et al., 2017). Pharmaceutical inhibition of mTOR has increased autophagy, reduced neuronal loss and cell death, and improved locomotor function after SCI in mice (Kanno et al., 2012). These pathways highlight the complexity of the secondary injury phase of SCI, the inter-linked nature of the responses and the prominent role of the inflammatory response after traumatic injury to the CNS.

#### The Inflammatory Response Changes With Age: ‘Inflamm-Aging’

The gradual increase in inflammatory signaling documented with age has been termed ‘inflamm-aging’ (De Martinis et al., 2005). ‘Inflamm-aging’ is largely microglia-driven; however, the accumulated effects are seen on a wide variety of cells and can fundamentally alter these cells’ normal physiological behavior. There are empirical links between inflammation and typical features of aging brains, such as decreased re-myelination, gray matter loss and cortical thinning, shrinkage in hippocampal volume, and deficits in learning and memory (Koellhoffer et al., 2017). It has also been suggested that age-related increases in ROS lead to chronic inflammation. Microglia are responsible for the bulk of oxidation products and inflammatory mediators in the aged brain, and contribute to a gradual increase in ROS generation with age. They are also key mediators of ROS-induced neural injury and ROS contribution to neurodegeneration (see Stewart et al., 2019 for review). NADPH-oxidase (NOX) has been linked to the classical activation of microglia and demonstrated to contribute the bulk of superoxide in neurodegenerative disorders (Bordt and Polster, 2014). NOX levels have been seen to increase in the CNS with age. Increased production of ROS from macrophage NOX with age exacerbates secondary injury after SCI (Zhang B. et al., 2019). Dystrophic microglia in aging have been observed to contain degenerated mitochondria (Wierzba-Bobrowicz et al., 2004). When stimulated with lipopolysaccharide (LPS) microglia that are deficient in NOX exhibited decreased tumor necrosis factor (TNF) expression, decreased levels of intracellular ROS and extracellular superoxide, and subsequent decreases in neurotoxicity (Qin et al., 2004). The increase in ROS production in the aging CNS can be directly toxic to adjacent neurons and can influence them via secondary signaling pathways, such as the NFκB or MAPK pathways.

### Macrophages, Microglia and Phagocytes in the Central Nervous System

Microglia play an important role in the development, monitoring and maintenance of the CNS. These cells, alongside peripheral monocytes/macrophages, also have a significant role in the post-injury environment (Fleming et al., 2006; Benowitz and Popovich, 2011). The dichotomy of macrophages and microglia stems from the heterogeneity of these cells. Their different activation phenotypes play different roles in the potential deterioration or repair of the surrounding tissue (Kigerl et al., 2009; Murray and Wynn, 2011), especially in relation to neuronal survival and axon growth. Activated microglia and macrophages have been suggested to both promote (Rabchevsky and Streit, 1997) and inhibit (Popovich et al., 2002) neurite outgrowth under different conditions (Gensel et al., 2009).

The functional states of macrophages are broadly categorized along a spectrum between classically activated pro-inflammatory (M1-like) and alternatively activated anti-inflammatory macrophages (M2-like) (Kigerl et al., 2009; Gensel and Zhang, 2015). Classically activated macrophages are efficient producers of potentially neurotoxic effector molecules and pro-inflammatory cytokines, while the alternatively activated phenotype is involved in dampening the inflammatory response and promoting tissue remodeling (Mantovani et al., 2013). While in the adult brain microglial density remains fairly stable over a lifetime, there is constant steady turn-over of microglia (cells surviving an average of 4.2 years) (Tan et al., 2020), allowing for the population to be refreshed and renewed multiple times in a lifetime (Askew et al., 2017).

#### The Microglial and Macrophage Response to Injury in the Young Nervous System

The microenvironment of the lesion after SCI affects the function and polarization of macrophages (Brennan and Popovich, 2018). Constituents of the injury microenvironment, including myelin debris, can switch phagocytes toward a classically activated phenotype, propagating the pro-inflammatory response (Brennan and Popovich, 2018). The temporal distribution and the magnitude of pro-inflammatory and anti-inflammatory responses after SCI may have a significant role in determining the efficacy of injury resolution (Gordon and Martinez, 2010). Classically pro-inflammatory macrophages are prominent in the initial inflammatory response to SCI, persist at increased levels (Donnelly and Popovich, 2008), which can be detrimental to injury resolution. The transient nature of the anti-inflammatory macrophage response, and its lower magnitude (Gordon and Martinez, 2010; Sutherland et al., 2017) contribute to the persistence of pro-inflammatory responses, and the development of a neurotoxic inflammatory state (Ren and Young, 2013). This translates into poor neural tissue regeneration and wound resolution, resulting in poorer functional recovery.

Macrophages and activated microglia in contact with axons at the site of a CNS injury promote ‘dieback’ of axons from the lesion, signaled through both cell surface molecules and released soluble factors (Horn et al., 2008; Busch et al., 2011). Macrophages activated by intra-spinal zymosan displayed concurrent pro-regenerative and neurotoxic functions in a non-traumatic model (Gensel et al., 2009). This model showed a significant increase in axon growth up to the foci of activated macrophages. However, the axon growth was transient and the neurotoxic effects resulting from prolonged exposure to inflammatory factors produced by the activated macrophages persisted, resulting in impaired axon growth and increased cell death. LPS elicited a strong pro-inflammatory response without any accompanying axon growth enhancement, further demonstrating the heterogeneity of macrophage responses (Gensel et al., 2009).

Despite the evidence suggesting a negative effect of microglia on neuronal regeneration after CNS trauma (reviewed in Silver et al., 2015), these cells are essential to the CNS. The complete depletion or blocking of microglia after injury has been shown to exacerbate injury pathology and impair recovery (Lalancette-Hébert et al., 2007). Non-specific immune therapies are largely ineffective and can often worsen the injury outcome (Brennan and Popovich, 2018). After traumatic injury microglia and macrophages are capable of producing neurotrophic factors such as brain-derived neurotrophic factor (BDNF) (Dougherty et al., 2000) and the macrophage derived protein oncomodulin (Yin et al., 2006) that can promote axonal growth. In the post-injury environment pro-inflammatory modulators prevail. Pro-inflammatory cytokines play a necessary role when kept in balance, and a complete blockade of their activity may be detrimental to recovery (Ikeda et al., 1996; Streit et al., 1998). Both pro- and anti-inflammatory effectors are required; however, the response needs to be regulated and the magnitude and timing closely controlled. It is this balance and regulation that is currently missing in the inflammatory response to CNS injury.

#### Altered Phagocyte Responses in the Aging Nervous System

In recent years, it has been recognized that a persistent mild pro-inflammatory state in elderly patients is correlated with significant degenerative diseases (Howcroft et al., 2013). The relationship between systematic inflammation and neuroinflammation in aged subjects is still unclear. Evidence indicates that peripheral activation of the innate immune system in aged animals leads to exacerbated neuroinflammation in the CNS (Godbout et al., 2005; Howcroft et al., 2013). It has been demonstrated in mice that activation of the peripheral innate immune system by LPS can induce CNS microglia to produce pro-inflammatory cytokines, resulting in reduction in locomotor and social behaviors (Godbout et al., 2005). The complement pathway, and molecules associated with microglial activation, are up-regulated in the naive aged spinal cord, suggesting that a para-inflammatory state develops with aging (Galbavy et al., 2017).

With aging cellular senescence, dysregulated inflammatory signaling and defects in phagocytosis can result in maladaptive immune responses, chronic inflammation, and worsened outcomes after injury (Streit et al., 2008; Koellhoffer et al., 2017; Ritzel et al., 2019). PNS axons recover more efficiently when debris is cleared; however, aged Schwann cells exhibit diminished plasticity, and impairments in clearance of myelin debris as well as in recruitment of other phagocytes (Painter et al., 2014). In the aging brain the number and density of microglia increase in the parenchyma of various compartments, however, the reason for this increase is still unclear (Mouton et al., 2002). The distribution of microglia is altered in the aging cortex, moving away from a uniform, organized ‘mosaic’ to a less even distribution (Tremblay et al., 2012). Additionally, the normal ramified appearance of microglia shifts toward a de-ramified appearance with shorter and less branched processes (Damani et al., 2011). This is likely to have detrimental functional consequences as the microglial processes are dynamic structures important to the surveillance and support functions of microglia (Wong, 2013). Aged microglia are considered ‘primed’ and have exaggerated responsiveness to acute inflammatory stimulus (O’Neil et al., 2018) as well as systemic inflammation (d’Avila et al., 2018). Age-associated changes to microglia have been implicated in the progression of neurodegenerative disorders, such as Parkinson’s (Lecours et al., 2018) and Alzheimer’s (AD) (Hickman et al., 2008) diseases, and their prevalence in aging populations documented (Wong, 2013).

A beneficial inflammatory response to injury is reliant on correct regulation, magnitude, and balance between pro-and anti-inflammatory impetuses. Inhibitory receptors on microglia are required to prevent the initiation of undesirable inflammation and to resolve inflammation. In aged nervous tissue these receptors show an impaired ability to maintain microglial quiescence, which may be partially due to a reduction in the expression of the receptors’ cognate ligands (Koellhoffer et al., 2017). Two examples shown to be impaired in the aged CNS are the immune-inhibitory signaling axes CD200/CD200R1 (Wang X. J. et al., 2011) and CX3CL1/CX3CR1 (Wynne et al., 2010). Both of these are associated with downregulating microglial activation and maintaining homeostasis in the CNS. The CD200/CD200R signaling axis is involved in microglia retaining a quiescent state. In the aging brain, there is a decreased of CD200 expression at both the mRNA and protein levels, accompanied by an enhancement of microglial response (Wang X. J. et al., 2011). CX3CL1/CX3CR1 signaling, which is involved in modulation of microglia activity, is impaired in the aging brain and CX3CL1 protein levels are reduced. Specifically in isolated microglia, there is a decrease in CX3CR1, with a concurrent increase in pro-inflammatory cytokine IL-1β. There is also down-regulated surface expression of CX3CR1 on a subset of aged microglia, compared to their younger adult counterparts. This may be a key contributor to the exaggerated microglial response to LPS challenge observed and correspond to delayed recovery from sickness behavior, and prolonged IL-1β induction in the aged brain (Wynne et al., 2010). The limited lifetime turnover of microglia leaves them vulnerable to age-related deficits. Forced microglial repopulation in the aged (16–18 months) mouse brain reversed age-related deficits; however, the age-associated inflammatory signature in the whole brain was not rescued by microglial repopulation alone (O’Neil et al., 2018). Similar to O’Neil et al. (2018) another study found that microglial repopulation did not broadly alter the inflammatory gene expression profile of the brain, or the response to immune challenges. There were, however, reversals of age-related alterations in neuronal gene expression profiles and neuronal complexity, accompanied by rescue of deficits in long-term potentiation in aged neurons (Elmore et al., 2018). This study observed improvements in spatial memory and in physiological characteristics of microglia in 24 months old mice within a month of microglial repopulation. These studies suggest that microglia can be targeted to ameliorate some age-related deficits but they cannot be separated from the complex post-injury environment.

There have been studies demonstrating that aged microglia contribute to enhanced pathology and worse outcomes in traumatic brain injury (TBI) (Ritzel et al., 2019), Stroke (Moraga et al., 2015), and AD (Hickman et al., 2008). In SCI, macrophages and microglia play an important role in the secondary injury phase. The age-related differences in the pro-inflammatory properties of the endogenous microglia is thought to be a potentially important contributor to the more detrimental inflammatory response seen in adult animals (Kumamaru et al., 2012). Both aging and CNS trauma prime microglia and the inflammatory response, and as such, each can exacerbate the other. Aged individuals have been shown to have worse post-TBI outcomes, which may be associated with not just inflammation but also endocrine disfunction. Endocrine disruption is common in TBI, potentially stemming from hypotension, hypoxia, anemia, and inflammation in the secondary injury (Ziebell et al., 2017). Together these studies suggest that age exacerbates microglial activation, and inflammatory gene expression, detrimentally in CNS injury.

**FIGURE 3:**
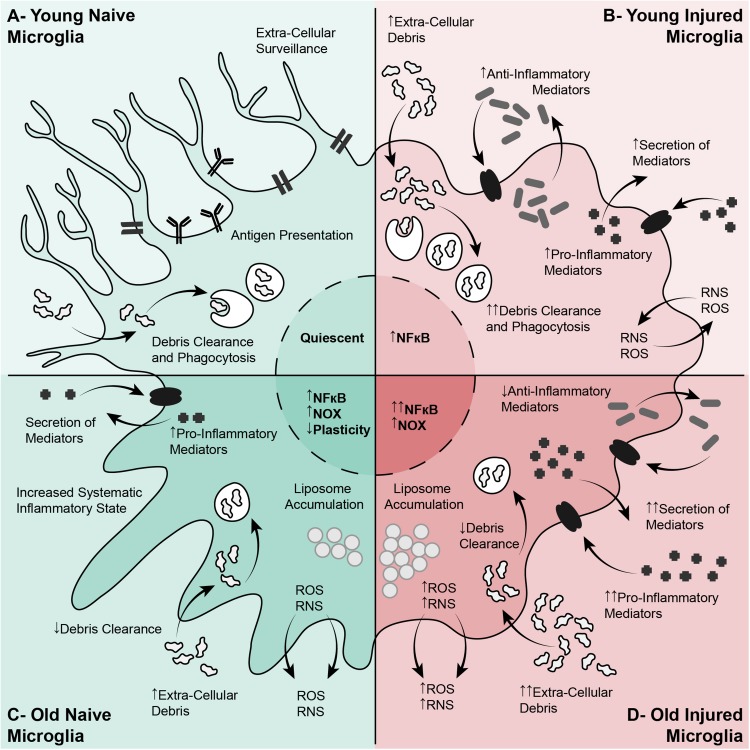
Alterations in microglia in aging, and their responses to spinal cord injury, exacerbate the injury and reduce axon growth. **(A)** Young Naïve Microglia. In the young naïve CNS, resting microglia have a ramified appearance with branched processes, and have an organized distribution. These cells are involved in extracellular surveillance, debris clearance, environmental maintenance, and antigen presentation. **(B)** Young Activated Microglia. In the young CNS, microglia are activated after a SCI, becoming more ameboid and phagocytic. This is characterized by increased debris clearance, upregulation of inflammatory mediators (mostly pro- and some anti-inflammatory), activation of inflammatory signaling pathways, and the production of ROS and RNS. **(C)** Aged Naïve Microglia. In the aging CNS, microglia undergo physiological and functional changes linked with a systematic para-inflammatory state. Aged microglia have a de-ramified appearance and a more disorganized distribution. These cells are less plastic and become ‘primed,’ characterized by increased expression of pro-inflammatory mediators, increased NOX, ROS and RNS, impaired debris clearance, and liposome accumulation. **(D)** Aged Activated Microglia. After a SCI in the aged CNS, aged microglia respond similarly to the younger microglia. However, these aged cells are already ‘primed,’ pro-inflammatory, and have impaired debris clearance. This results in exacerbation of the detrimental elements of microglial activation, such as excessive production of pro-inflammatory mediators into the lesion environment, excessive NOS, ROS and RNS leading to increased oxidative stress, and inefficient debris clearance. CNS, Central Nervous System; NOX:, NADPH-oxidase; ROS, Reactive Oxygen Species; RNS, Reactive Nitrogen Species; SCI, Spinal Cord Injury.

With age, phagocytosis and debris clearance by macrophages and monocytes declines (Linehan et al., 2014). Deficits in phagocytosis have also been observed in the normal and challenged CNS in aged animals (Neumann et al., 2008; Natrajan et al., 2015; Ritzel et al., 2015). The fragmentation of the myelin sheaths increases with age and leads to the formation of insoluble lysosomal inclusions in microglia, contributing to age-related microglial senescence and immune dysfunction (Safaiyan et al., 2016). The increased myelin debris in the aged CNS also affects the phenotype of phagocytes in CNS injury. Infiltrating macrophages often present an anti-inflammatory phenotype upon entering the CNS and migrate toward the injury epicenter where they switch to a more pro-inflammatory phenotype. Anti-inflammatory primed blood-derived macrophages respond to myelin debris in culture, shifting to resemble more classically activated macrophages (Wang et al., 2015). In a model of demyelination, aged macrophages and microglia showed excessive accumulation of myelin debris which led to cholesterol crystal formation, phagolysosomal membrane rupture and stimulated inflammasomes (Cantuti-Castelvetri et al., 2018). The alterations in microglia with injury and aging are shown in Figure 3. The interactions between the inhibitory factors in the lesion, and the macrophages, microglia and astroglia, creates a largely neurotoxic and inhibitory environment post-injury, exacerbated in aging.

### ‘Inflamm-Aging,’ Axon Growth and Recovery From Spinal Cord Injury

Inflammation is viewed as an important driver of age-related CNS dysfunction. There is very little known about how the changes in the inflammatory response that occur with age influences the response to SCI. The literature in this area is sparse. Both microglial and astroglial responses are increased and more sustained in older animals, however, aged microglia may be less efficient at debris clearance (Li, 2013). Up to 30 days post-injury there is a greater increase in phagocytes after contusion SCI in middle aged rats, compared to their younger counterparts. Older animals exhibited a significant increase in CD68-positive cells and demonstrated a more pronounced sub-acute CD68 upregulation around the injury site (von Leden et al., 2017). In a thoracic SCI study greater locomotor deficits have been observed in aged (18 months) animals compared to their younger (3 months) counterparts (Hooshmand et al., 2014). The lesion size and amount of cell death was increased, with much lower tissue sparing in the aged animals. The acute localized inflammatory response was also significantly higher. Microglial markers and activation have been observed to increase with age, however, the function of these activated microglia is still largely unknown as is whether this increase is causative or reactive to neurodegeneration (Howcroft et al., 2013). The affect the aging inflammatory response has on recovery from SCI is an area that needs greater attention with the aging SCI population.

Age-related differences in inflammation have been explored to some extent in infants and younger adult animals, particularly in rodents (Sutherland et al., 2017) but have yet to be explored in middle-aged and aging animals. It is believed that the alternative activation of either microglia or macrophages to a M2a phenotype by interleukin (IL)-4/IL-13 may support neuronal growth and repair processes (Kigerl et al., 2009; Murray and Wynn, 2011). Aged (18–19 months old) mice have demonstrated impaired induction of the IL-4α receptor (IL-4Rα) in microglia, accompanied by attenuated downstream Arginase gene expression (anti-inflammatory associated), as well as decreased recruitment of IL-4 receptor expressing macrophages. Aged mice, both wild-type and IL-4Rα knockout, also displayed decreased functional recovery after contusion SCI (Fenn et al., 2014). Macrophages in 14 months old mice exhibited significantly reduced IL-10 expression and CD86/IL-10 (M2b) macrophages in the spinal cord after spinal contusion. This decrease in M2-like phenotype coincided with increased tissue damage and greater functional impairment (Zhang et al., 2015). In a contusion SCI study in mice significantly higher generation of ROS occurred in 14 months old mice compared to their 4 months old counterparts. This was accompanied by NOX2 increases and increased indications of lipid peroxidation. This suggests that macrophages and NOX contribute to SCI oxidative stress that is exacerbated in aging (Zhang B. et al., 2016). In middle-aged rats, both naïve and after a moderate contusion SCI, there were significant increases in markers of oxidative stress, microglial marker Iba1, and inflammatory and NOX2 component gene expression compared to their younger counterparts. These middle-aged rats also exhibited greater lesion volume and showed significantly reduced hind-limb motor scores after the contusion SCI (von Leden et al., 2017). These studies suggest the aged inflammatory state is more detrimental to the surrounding tissue and inhibitory to ongoing regeneration and repair. Both aging and spinal cord injury prime microglia, produce a pro-inflammatory environment and a detrimental oxidative environment, it follows that in combination these two factors will compound one another to worsen recovery after SCI.

## Astrogliosis and the Astroglial Scar in the Central Nervous System

### Normal Role and Functions of Young Astrocytes

Astrocytes have a broad range of roles in the development and maintenance of the CNS. In the mature CNS, they are involved in the cellular, molecular and metabolic homeostasis essential for normal neuronal function. Astrocytes monitor and maintain the extracellular ionic environment and pH, as well as clear extracellular glutamate and gamma aminobutyric acid (GABA). It has emerged that astrocytes also play a role in detoxifying free radicals to prevent excitotoxicity. These cells also have a role in synthesizing precursors for glutamate and GABA production. Furthermore, astrocytes have been shown to sequester and redistribute potassium (K^+^) for neuronal activity. These glia cells produce metabolic substrates for neuron function, including lactate and the production and release of ATP. Astrocytes store glycogen and can produce energy substrates to subsist on anaerobic ATP production. For in depth review see Ransom et al. (2003). Astroglia are also involved in the maintenance of the blood-brain barrier (BBB) and can release factors that modulate endothelial permeability (Abbott, 2002) allowing for the exchange of essential nutrients into the CNS. The processes of astrocytes are associated with capillaries and involved in maintaining cerebral blood flow to support neuronal activity (Mulligan and MacVicar, 2004). Additionally, astrocytes can regulate brain microvascular permeability via Ca^2+^ signaling (Erdő et al., 2017).

Astrocytes are also involved in the sculpting, maintenance and modulation of excitatory and inhibitory synapses and regulation of synaptogenesis (Ransom et al., 2003). They produce factors to induce synapse formation and maturation, and are involved in ‘synaptic pruning’ in the developing nervous system. In the functional synapse perisynaptic astrocytic processes house transporters and enzymes responsible for ion and transmitter homeostasis in the synaptic cleft, as well as for metabolic support (Pekny et al., 2016).

### Alterations in Astrocytes in Normal Aging

Astrocytes are vital to homeostasis, defense and regeneration of the CNS. In the aging brain, there are alterations in astrocyte morphology, and loss of functionality and reactivity. These changes are heterogeneous, disease-related, and region-specific (Clarke et al., 2018; Cunningham et al., 2019; Matias et al., 2019). In aging the brain, astrocytes play a role in the hypometabolic state that impinges on mitochondrial metabolism and neuronal survival (Jiang and Cadenas, 2014). With age, astrocytes exhibit an early stage of reactive astrogliosis, even in the absence of disease or trauma (Cotrina and Nedergaard, 2002; Boisvert et al., 2018). This is observed in cellular hypertrophy and variations in the expression of structural elements, such as glial fibrillary acidic protein (GFAP) and vimentin, and growth factors, such as ciliary neurotrophic factor (CNTF). An increase in oxidoreductive enzymes is also observed (Cotrina and Nedergaard, 2002). These are hallmarks of reactive astrogliosis that are seen in pathological conditions in the younger CNS. Similar astrocytic changes have been found in the aging spinal cord (Duan et al., 2009). There is evidence that astrocytes undergo cellular senescence with aging. The increase in neuroinflammatory function and loss of normal homeostatic function associated with astrocytic senescence may be an important component of neurodegenerative disorders prevalent in aging (Cohen and Torres, 2019). It has also been observed that the number of astrocytes in some brain regions, especially the cortex, increase significantly with aging (Cotrina and Nedergaard, 2002). Astrocytes in aging are extensively reviewed in Palmer and Ousman (2018).

The physiological changes in astrocytes with aging affect ability to perform their core functions, and can contribute to BBB disfunction (Erdő et al., 2017), alterations in antioxidant systems and increases in oxidative stress (Pertusa et al., 2007; Duan et al., 2009), an increased and altered inflammatory state (Clarke et al., 2018), and reduced neuronal support (Das and Svendsen, 2015). Age-dependent astrocytic alterations may affect synaptic efficacy in normal tissue and also hinder neuronal survival in pathological conditions. Aged astrocytes have been shown to upregulate genes that partially resemble reactive astrocytes, and also those associated with synapse elimination, that varies region to region within the brain. These alterations in aged astrocytes result in an environment susceptible to neuronal damage, a potential factor in age-related diseases and decline (Boisvert et al., 2018). Astrocytic differences in aging animals may impact the efficiency of post-SCI astrogliosis, specifically, the ability of reactive astrocytes to quickly sequester the inflammatory response and reduce its spread, which may result in worse outcomes for patients. One possibility is that a greater imbalance between the protective effects and the neuro-inhibitory effects of reactive astrogliosis develops with aging. Astrocytes undergo an increase in basal mRNA levels of activated astrocyte markers GFAP and S100B in the aged brain (Popa-Wagner et al., 2011). In the normal brain, the region-specific transcriptional identities of astrocytes change with age and astrocytes in the aged brain show the neuroinflammatory phenotype of A1-like reactive astrocytes (Clarke et al., 2018). The differences in astrocyte properties and reactive astrogliosis that occur with age is not a new question, with comparative studies as far back as the 1980s, however, it is still an area with many unanswered questions. In a cortical/hippocampal needle-puncture model, both 3 and 16–19 months old rats showed robust early astrocyte proliferation. This response was heterogenous and regional, with the older rats demonstrating greater proliferation in the cortex not in the hippocampus (Topp et al., 1989). After chemically induced excitotoxity in the striatum aged (22–24 months) rats exhibited an earlier onset and a more pronounced astrogliosis than young (3 months) rats. This is correlated with a delayed progression of neurodegeneration in the aged striatum, and reduced tissue injury and edema size (Castillo-Ruiz et al., 2007).

Reactive astrogliosis and astrogliopathy play an important role in diseases of the nervous system, especially neurodegenerative diseases, that increase in prevalence with age. In aging-prevalent diseases, such as AD, tauopathies, Lewy body diseases, Huntington’s disease (HD), amyotrophic lateral sclerosis (ALS) and Creutzfeldt-Jakob disease, astrogliopathy is often characterized by increased GFAP and abnormal protein aggregates (Ferrer, 2017). After cerebral ischemia, astrocytes form an astroglial scar around the infarct. In a stroke model, aged rats (18–24 months) demonstrated more severe functional impairment, accompanied by an accelerated glial response, compared to their young (2 months) counterparts. This suggests alteration in the temporal progression of the glial response with age may result in altered scar formation and an environment that is more inhibitory to axon growth (Badan et al., 2003). After stroke, levels of the immune modulator transforming growth factor (TGF)-β are increased in astrocytes, activated microglia and macrophages, suggesting that this increase may be associated with regulation of the astroglial scar formation and the immune response. TGF-β is involved in the maintenance of homeostasis, especially vascular homeostasis (Walshe et al., 2009), therefore, an increase in astrocytic TGF-β may suggest a deviation from homeostasis and vascular dysfunction. While TGF-β signaling is increased in the aged brain, it is yet to be correlated with infarct size or functional outcomes (Doyle et al., 2010). In a rodent model of ALS, an age-dependent acceleration in acquiring a senescent phenotype has been observed in astrocytes, accompanied by a reduction in their support of motor neurons (Das and Svendsen, 2015).

### Trauma and Reactive Astrocytes in the Young Nervous System

One of the hallmarks of trauma to the CNS is reactive astrogliosis, and the formation of a glial and fibrotic scar (Orr and Gensel, 2018). Reactive astrogliosis is the molecular and morphological changes that astrocytes undergo (Sofroniew, 2009). Astrocytes become reactive and undergo functional and physiological changes in trauma, disease and aging (Matias et al., 2019). These alterations include proliferation, hypertrophy of the soma and processes, branching, and upregulation of GFAP expression, and occur in a heterogeneous, context dependent manner regulated by different signaling events (Sofroniew and Vinters, 2010; Anderson et al., 2014). In severe cases reactive astrogliosis results in the formation of the astroglial scar. This is an important process after injury as these cells wall off the damaged areas and seal the lesion to protect the surrounding intact tissue from further damage (Faulkner et al., 2004). Indeed, removal of reactive astrocytes after neurotrauma results in failure of BBB repair, widespread tissue disruption, pronounced cellular degeneration, and severe motor deficits (Faulkner et al., 2004), indicating the role of reactive astrocytes in restoration of the BBB. However, there is still some contention as to whether reactive astrogliosis, and the formation of the astroglial scar, are beneficial or detrimental to functional recovery after SCI (Fawcett and Asher, 1999; Silver and Miller, 2004; Sofroniew, 2009). The role of astrocytes and microglia in regenerative failure in the CNS has been extensively reviewed previously (Silver et al., 2015).

There have been many studies demonstrating that reactive astrocytes have a substantial effect on axon regeneration after SCI. *In vitro* studies have shown that activated astrocytes produce both pro- and anti-inflammatory effector molecules, which can both help and hinder functional recovery (Sofroniew, 2009; Kawano et al., 2012; Pekny and Pekna, 2014). The astroglial scar is not only a physical barrier, obstructive to axon growth, it also produces a variety of potent growth inhibitory ECM molecules, and the lectican family of chondroitin sulphate proteoglycans (CSPGs) (McKeon et al., 1991, 1999; Li et al., 2013). For decades, the glial scar has been acknowledged as a source of molecules which can inhibit neurite outgrowth (Bovolenta et al., 1992). A 2011 study also suggested that reactive astrocytes also contribute to the failure of re-myelination by high expression of bone morphogenic protein (BMP) (Wang Y. et al., 2011). A study in adult mice demonstrated that neither complete prevention of the astroglial scar formation, attenuation of reactive astrocytes, or the deletion of chronic astroglial scars resulted in increased spontaneous growth of severed axons through a thoracic SCI. Even with the introduction of axon-specific growth factors, preventing the formation of the astroglial scar still significantly reduced the stimulated axon (Anderson et al., 2016).

Recently reactive astrocytes have been shown to be a highly heterogeneous population (Zamanian et al., 2012; Pekny and Pekna, 2014). Neuroinflammation and ischemia induce two different types of reactive astrocytes, referred to as ‘A1’ and ‘A2’ astrocytes respectively. A1 astrocytes demonstrate a more detrimental neuroinflammatory profile whereas A2 astrocytes upregulate many neurotrophic factors and appear more beneficial to injury resolution (Zamanian et al., 2012). A1 reactive astrocytes exhibit impaired ability to carry out normal astrocytic functions, and also release neurotoxic elements that may contribute to damage (Clarke et al., 2018). This heterogeneity of the reactive astrocyte population may go some way to explaining the dichotomy of results around reactive astrogliosis after CNS injury. In the field of SCI there is a large body of literature demonstrating both neuro-protective and neuro-inhibitory elements of post-traumatic reactive astrogliosis (Fawcett and Asher, 1999; Silver and Miller, 2004; Sofroniew, 2009). The duality of the astroglial scar and reactive astrogliosis is still a complicating factor in potential treatments for SCI, which is further impacted by changes in the aging CNS. This is an interesting path to explore in the context of the astrocytic response to SCI and also how this is altered with age.

In the astrocytes, several molecular pathways have been shown to be involved as regulator of reactive astrogliosis in trauma and disease. Studies have pointed to the Janus Kinase (JAK)/STAT3 signaling as an important pathway (Okada et al., 2006; Herrmann et al., 2008). In models of AD and HD the over-expression of SOCS3 in astrocytes inhibited the activation of the JAK/STAT3 pathway and attenuated astrocyte reactivity (Haim et al., 2015). Astrocytic *STAT3* deletion reduces GFAP upregulation and scar formation after SCI, as well as increased spread of inflammation, tissue disruption and locomotor deficits (Herrmann et al., 2008). Conversely, astrocytic *SOCS3* deletion, an endogenous inhibitor of STAT3, results in rapid astrocyte migration, deceased lesion area and better motor outcomes (Okada et al., 2006). Activation of the PI3K/Akt/mTOR signaling pathway is involved in astrogliosis. In culture, mTOR induces astrocyte proliferation, and its blockade suppresses proliferation, attenuates astrocytic migration, and reduces production of astrocytic-induced inflammatory factors (Li et al., 2015). Overexpression of *PTEN* in astrocytes attenuates astroglial scar formation, alters CSPG expression and improves axon regeneration into the lesion site (Chen et al., 2016). However, the role of mTOR signaling in both the astrocytic and inflammatory responses, is yet to be fully elucidated. There are mixed reports as how the MAPK pathways are involved in astrogliosis. After traumatic SCI, proliferation of reactive astrocytes in the lesion is accompanied by an increase in the expression and phosphorylation of mitogen-activated protein kinase kinase-extracellular signal-regulated kinases (MEK-ERK). A specific increase in the level of ERK phosphorylation was detected specifically in astrocytes after SCI (Ito et al., 2009). Inhibition of p38 MAPK or ERK has been demonstrated to be protective ischemia or brain injury models (Kang and Hébert, 2011). In another study, inhibition of p38 MAPK appeared to worsen ischemic damage (Lennmyr et al., 2003). Recently, leucine zipper-bearing kinase (LZK) has been shown to mediate astrogliosis, as its deletion in astrocytes reduces astrogliosis after SCI, subsequently increasing the lesion size, while its over-expression enhances astrogliosis and decreases the lesion size (Chen et al., 2018). Together, these suggest that the role of the MAPK signaling in astrocytes response still needs to be elucidated. Others pathways such as NFκB may also be involved in astrogliosis (Kang and Hébert, 2011). NFκB, mTOR, JNK/STAT3 and MAPK pathways are all linked to not just astrocytes and reactive astrogliosis, but are also involved in the inflammatory and neuronal response, demonstrating the complex interlinking of extrinsic factors as well as neuron-intrinsic factors after CNS trauma.

### The Aged Astrocyte Response to SCI

Astrocytes in aging exhibit a range of physiological changes and functional alterations that can impact their response to trauma. Variations in structural elements, growth factors, and oxidoreductive systems (Cotrina and Nedergaard, 2002) in naïve aged astrocytes can compound the existing neurotoxic elements of the injury micro-environment by increasing the detrimental oxidative state and decreasing support for neuron regeneration. The loss of antioxidant tolerance capacity and increased vulnerability to oxidative stress with age (Pertusa et al., 2007; Duan et al., 2009) may be a significant contributor to the increase in post-injury ROS, and oxidative stress after CNS trauma. Significantly, the increased inflammatory state, and skew toward pro-inflammatory phenotypes (Clarke et al., 2018) in aging astrocytes can have a significant impact on the response to trauma. *In vitro* aged astrocytes show amplified responses to inflammatory cytokines, IL-1β, and TNF-α (Jiang and Cadenas, 2014). The primed, early activation state of these cells (Cotrina and Nedergaard, 2002) may have a cumulative effect with the injury induced reactive astrogliosis, exacerbating the negative effects of the persistent pro-inflammatory response. This warrants investigation, especially in the field of SCI.

There have been few studies comparing younger and aged astrocytes responses to SCI. In primary spinal cord astrocytes aged *in vitro* (60 DIV), increased expression of ferritin and GFAP were observed along with decreases in glutamate transporter 1 and transferrin receptor 1, and in mitochondrial membrane potential (Duan et al., 2009). Coincidently, in the naïve middle-aged (13 months) mouse spinal cord, decrease in antioxidant enzymes heme oxygenase 1 and NAD(P)H/quinone oxidoreductase 1, and in nuclear factor E2-related factor 2 (Nrf2) which directs expression of these enzymes, were observed (Duan et al., 2009). *In vitro*, aged astrocytes display an age-dependent increase in mitochondrial oxidative metabolism. The activation of NFκB signaling in these cells results in increased expression of NOX2 and elevated nitric oxide production. This was also associated with an age-dependent increase in hydrogen peroxide generation (Jiang and Cadenas, 2014). Alterations in the antioxidant capabilities and mitochondrial function may have significant flow-on effects in injury scenarios where there is oxidative damage and a detrimental increase in ROS and RNS. In response to SCI in older animals, astrocytes may also present changes in the production profile in CSPGs, and in their role in maintaining blood-spinal cord barrier (BSCB) integrity, which could have far-reaching consequences through the entry of inflammatory and potentially neurotoxic elements into the CNS and which also impact the availability of nutrients and support for potentially regenerative tissue after trauma.

In the area of SCI, the differences in astrocyte reactivity that occur in aging may have significant and far-reaching consequences. Aging astrocytes have been observed to lose their capacity to support neurite outgrowth and their efficacy stimulating axon growth (Smith et al., 1990). In the aging spinal cord, astrocytes exhibit higher basal GFAP mRNA levels, however, their ability to respond to a non-invasive CNS injury was observed to be reduced compared to their younger counterparts (Kane et al., 1997). This is potentially significant in the context of SCI as astrogliosis is an important element of the post-injury response and the interaction between astrocytes and axons is highly influential for axon growth potential. Recently, neuronal *PTEN* deletion was shown to promote mTOR signaling and axon growth in both the rubrospinal and corticospinal tracts, regardless of the age of the mice, but the axonal regeneration was still greatly diminished in the older animals compared to their younger counterparts (Geoffroy et al., 2016). An increase in astrogliosis, as well as inflammatory marker CD68, was observed in older animals at sub-chronic time-points, suggesting that differences in neuron-extrinsic environmental influences could mediate the age-dependent decrease in axon regeneration. It would be interested to assess whether modification of the astroglial scar with neuronal *PTEN* deletion could enhance axon regeneration in middle-age animals.

**FIGURE 4:**
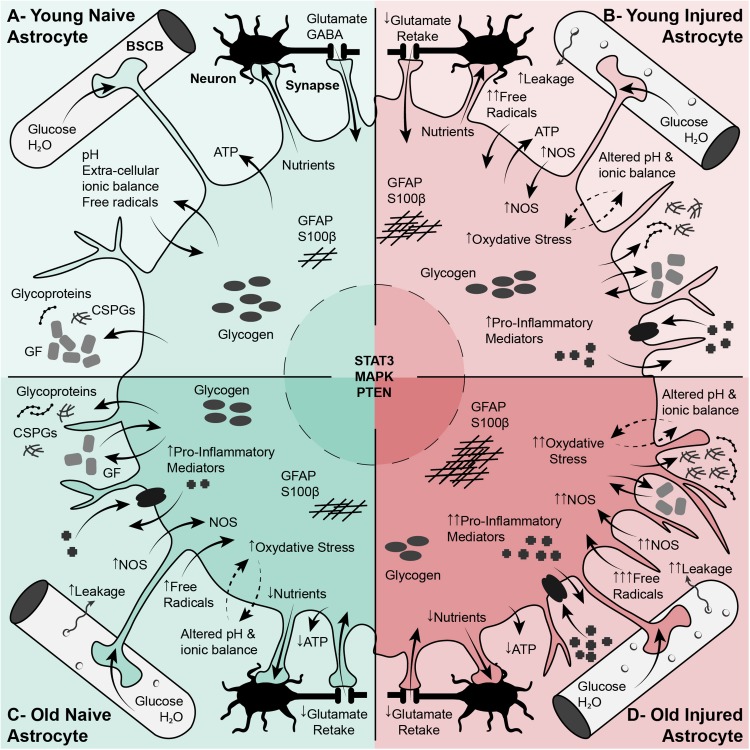
Differences in astrocyte phenotypes, functions and responses with aging play a significant role in central nervous system health as well as spinal cord injury response. **(A)** Regular Function of Young Astrocytes. Astrocytes play a vital role in cellular, molecular, and metabolic homeostasis in the naïve CNS. These cells are involved in synaptogenesis and support mature synapses. They also support neurons and neuronal function through the uptake of glutamate, K+, and GABA, the production and release of transmitters, cytokines and ATP, and by stocking glycogen. Astrocyte processes interact with vascular endothelial cells and are an important element of the BBB. **(B)** Young Reactive Astrocytes. After SCI, astrocytes become reactive, increasing in size and branching. These reactive astrocytes are characterized by a suite of molecular changes including increased GFAP expression, increased proteoglycan (CSPGs) production, increased NOS and ROS, and production of inflammatory cytokines. Reactive astrocytes are involved in BBB repair, and sequestering the inflammatory lesion and fibrotic scar. **(C)** Normal Aging Astrocytes. In the aging CNS, astrocytes become larger and more branched than their younger un-injured counterparts. These cells exhibit an early stage of reactive astrogliosis characterized by increased GFAP, vimentin and S100β expression, increased expression of growth factors (such as CNTF and TGF-β) and increased oxidoreductive enzymes and oxidative stress. These cells show impaired ability to perform normal functions such as BBB/vascular maintenance and neuronal support. **(D)** Aged Reactive Astrocytes. After SCI, the alterations in astrocytes with aging are exacerbated. Aged astrocytes are already in an early reactive state, this is compounded by injury and activation cues in the lesion environment. The result is greater increase in GFAP, increase in oxidative stress and inflammation, increased excitotoxicity, impaired BBB repair, and, significantly, impaired sequestering of the inflammatory lesion. All of this will have profound effects on neuronal survival and axon re-growth. BBB, Blood-Brain Barrier; CNS, Central Nervous System; CNTF, Ciliary Neurotrophic Factor; CSPGs, Chondroitin Sulfate Proteoglycans; GFAP, Glial Fibrillary Acidic Protein; NOS, Nitric Oxide Synthase; ROS, Reactive Oxygen Species; SCI, Spinal Cord Injury.

Reactive astrogliosis and the formation of an astroglial scar are significant parts of the post-injury response in the CNS and can influence outcomes after injury. The glial response contributes significantly to the nature of the post-injury environment and may be an important extrinsic factor in regulating axon regeneration, however, this is an area that still needs to be examined in detail. Astrocytes exhibit changes in reactivity and response in the aging CNS that can have significant consequences for the progression of injury, and the development of CNS disorders. The significant alterations in astrocytic response with injury and aging are shown in Figure 4. This is potentially an important factor and one that we have little knowledge about. In the aging CNS these responses are robust but also irregular, which may contribute to the age-related decline in axon regeneration and also to worsening post-injury outcomes after SCI. This potential relationship between aging, the glial response and injury resolution is an area of great interest that requires a considerably more study.

## Cellular Influences Beyond Inflammation and Astrogliosis

While a great deal of attention and therapeutic efforts are directed at axon regeneration, with increasing focus falling on the inflammatory response and reactive astrogliosis, the secondary injury environment is potentiated by not just astrocytes and microglia but also fibroblasts, pericytes, endothelial cells, and other cells present around the injury epicenter (summarized in Figure 2). However, the specific role of each cell type in the age-dependent regeneration decline remains to be determined.

### Fibroblasts

After SCI, fibroblasts from different origins proliferate and form the fibrotic scar, which is isolated from the spare tissue by the reactive astrocytes. For detailed review see Dias and Göritz (2018). Fibroblasts are a major source of fibronectin, collagen and other ECM components known to inhibit regeneration. Reducing the fibrotic scar can enhance axon regeneration and increase functional recovery (Ruschel et al., 2015; Dias et al., 2018) in young animals. However, while most studies aim to reduce the fibrotic scar, ablation of fibroblasts may compromise tissue integrity after injury, elements of the fibrotic scar may play supporting roles, and removing them acutely after injury may have negative effects (Göritz et al., 2011; Bradbury and Burnside, 2019). In non-nervous tissue, aging is known to alter fibroblasts (Varani, 2010). Different pathways are altered in skin and cardiac fibroblasts with aging, including a decrease in ERK and Akt expression, an increase in JNK and TGF-β/Smad signaling, which can alter their proliferation and production of ECM proteins. Therefore, it is likely that age affects the fibrotic response after SCI. There is a need to understand how age specifically modify this response.

### Endothelial Cells

The vascular and nervous system are closely juxtaposed, and endothelial cells respond to similar patterning cues as growth cones (Adams and Eichmann, 2010). There is increasing interest in taking advantage of this interaction to control axon regeneration by controlling vascular patterning after SCI (Partyka et al., 2019; Tran et al., 2020). Endothelial cells secrete vascular endothelial growth factor (VEGF), which promotes cell survival and axonal outgrowth (Sondell et al., 1999). These cells can also secrete other neurotrophic factors promoting axon growth (Grasman and Kaplan, 2017). With age, the production of different factors by endothelial cells, such as VEGF, declines with age (Shetty et al., 2005), which can result in a decrease in cell survival and in axon growth with age. The production of endothelin-1 and nitric oxide by endothelial cells are also altered, resulting in the modification of vascular tone (vasoconstriction/vasodilation). Lack of control of the vascular tone can be result in inability to meet the metabolic demand of the tissue, increase of local hypoxia, and reduction in waste removal (Bosetti et al., 2016; Jacob et al., 2016). Alteration of this balance at the injury site is likely to have negative effect on axon regeneration. Finally, changes in vascular permeability and BBB leakiness with aging can result in infiltration of molecules into the CNS, alteration in the microenvironment, and increase in neurotoxicity. This demonstrates the need to better understand how SCI and age can impact endothelial cell function and their relation to axon growth potential.

### Pericytes

Pericytes are closely associated with endothelial cells, and have projections encircling CNS vessels. These cells not only play a role in regulating and maintaining vascular barrier integrity (Sá-Pereira et al., 2012), they have a wide range of physiological functions (Muramatsu and Yamashita, 2014). CNS trauma often leads to vascular disruption and dysfunction, closely linked to neuronal dysfunction. One significant element of this is the reduction in trophic support for the CNS cells. After SCI, excess of monoamine receptors (5-HT1) on pericytes leads to local constriction of capillaries and chronic hypoxia. Inhibiting this receptor increases locomotor recovery (Picoli et al., 2019).

Pericytes have emerged as potential targets to increase functional recovery after SCI. For a detailed review, see Picoli et al. (2019). Pericytes in the spinal cord are heterogenous. After SCI, a subtype of pericyte gives rise to a population of stromal cells integral to the glial and fibrotic scars, and play a role in post-injury fibrosis. These cells were shown to be essential to the successful sealing of the lesion (Göritz et al., 2011). Inhibiting the proliferation of pericytes reduces the fibrotic scar tissue after SCI, and improves the regeneration of axon tracts, prompting improved sensorimotor functional recovery (Dias et al., 2018). After SCI, pericytes contribute a significant amount of collagen to the scar formation (Birbrair et al., 2014) and NG2+ pericytes are also involved with angiogenesis (Hesp et al., 2018).

To our knowledge, how the age factor impacts the response of pericytes in SCI models has not been characterized. Pericyte loss and neurovascular dysfunction have been linked to age-dependent neurodegeneration. Alteration of the BBB is observed in different neurogenerative conditions (ALS, HD) as well as neurotrauma (TBI and stroke). For review see Lendahl et al. (2019) and Li et al. (2019). Therefore, it is conceivable that SCI in older animals’ results in enhanced BBB breakdown, increase in serum proteins leakage to the spinal lesion, increase in neurotoxicity as well as worse functional outcome. Additionally, a poorly functioning vascular system will likely result in less nutrients reaching potentially growing axons. Modulating pericytes integrity and activity could be of therapeutic interest in the context of middle-age and age SCI.

### NG2+ Cell Population

Different cell types can express NG2 in the CNS. Here, we refer to NG2+ cells as OPCs. OPCs are present throughout the CNS. They react to injury by proliferating and migrating toward the lesion, and differentiating into oligodendrocytes and astrocytes (Nishiyama et al., 2009; Hackett et al., 2018). For review see Hackett and Lee (2016). Removing NG2+ cells (both OPCs and pericytes) results in astrocytic and fibrotic scar disruption, with reduction in scar density and less distinct borders (Hesp et al., 2018). This was surprisingly accompanied by axon growth into the lesion as well as persistent functional impairment. While previous studies show that NG2 proteoglycan inhibits axon growth, NG2+ cells themselves may facilitate growth (Hackett and Lee, 2016). NG2+ cells proliferation and differentiation is reduced (Sim et al., 2002; Psachoulia et al., 2009) and OPCs heterogenicity altered with age (Spitzer et al., 2019). NG2+ cells have been shown to maintain neuron survival, via reduction of IL-1β pro-inflammatory pathway in the hippocampus (Nakano et al., 2017). Additionally, intracellular pathways involved in OPCs differentiation and myelination include ERK/MAPK, PI3K/Akt/mTOR and Wnt/β-catenin (Gaesser and Fyffe-Maricich, 2016), all of which can be altered with aging. An attractive hypothesis would be that the reduction in NG2+ activity with age and injury participates to neuroinflammation and inducing neuron death, and the reduction in NG2 cells proliferation and differentiation results in an environment less favorable to axon growth.

## The Influence of Extracellular Components on Axon Regeneration Potential

### The Extra-Cellular Matrix

The importance of the ECM and extracellular elements of the environment in an age-dependent decline in regeneration potential is highlighted in a study by Kawano et al. comparing postnatal 7, 14, and 21 with adult mice (P60-80) (Kawano et al., 2005). Transected axons regenerated across the lesion only in P7; in P14 and older, the axons stop at the lesion site. The fibrotic scar, containing type IV collagen, was not present in the P7 mice. Inhibition of collagen synthesis in older mice prevented the formation of this scar resulting in a greater number of axons extending across the lesion. These elements are intrinsically connected to the cellular elements and the formation of CNS scar tissue, as well as influencing the nature of the injury microenvironment. Overall, there has been very little done to elucidate the changes in extracellular components in aging and how it plays a role in the reduction of axon regeneration potential; the changes with age and traumatic spinal injury is still yet to be elucidated.

#### Glycoproteins

##### Fibronectin

After CNS trauma, the glial scar surrounds the lesion site, accompanied by infiltration of plasma fibronectin, fibronectin secretion from various fibroblast-like cells and formation of a fibrotic scar (Farooque et al., 1992; Zhu et al., 2015). While fibrosis restores BBB integrity and restricts lesion spread, it also disrupts the CNS structural matrix and produces both a physical and biochemical barrier to axon growth (Kawano et al., 2012). Astrocyte-associated fibronectin participates in axon growth (Tom et al., 2004) but fibronectin aggregates reduce remyelination (Stoffels et al., 2013). Fibronectin is an early trigger of glial scar formation through the TGF-β/Smad signaling pathway, and increases inhibitory proteoglycan expression (Schachtrup et al., 2010). Fibronectin can also activate microglia and infiltrating macrophages (Milner and Campbell, 2003). Reduction of the fibrotic scar, via reduction of fibronectin, could be an interesting strategy to increase axon growth. While changes in fibronectin expression with age after neurotrauma are unknown, one could predict an increase in expression level, as fibronectin increases with age in other tissue (Kumazaki et al., 1993; Dorner et al., 1996; Labat-Robert, 2004). This could lead to an increase in astroglial and inflammatory response, as well as a more inhibitory fibrotic scar.

##### Collagen

Collagens expression levels are increased after CNS injury (Frisen et al., 1995; Zhu et al., 2015). Although not inhibitory in itself, collagen IV serves as a mesh for other inhibitory molecules to bind (Liesi and Kauppila, 2002). Inhibition of deposition of Collagen IV facilitates axon regeneration (Stichel et al., 1999). An increase in collagens within the SCI lesion induces astrocytic scar formation via the integrin pathway (Hara et al., 2017). Blockage of collagen interaction with astrocytes reduces astrocytic scar formation and promotes functional recovery. However, collagen concentration is known to increase with age (Uspenskaia et al., 2004). It will be interesting to test whether alteration of the collagen composition with age after SCI is linked to the increase in the reactive astrocytes and formation of the astrocytic scar observed.

##### Laminin

Laminins are widely express in the CNS, and play important roles, mostly via the integrin pathway (Nirwane and Yao, 2019). Astrocyte-produced laminins maintain BBB (by regulating pericyte differentiation), and the increase in laminin observed after TBI may be an attempt in restoring the BBB integrity (Tate et al., 2007; Yao et al., 2014). Laminins participate in angiogenesis and revascularization after injury (Simon-Assmann et al., 2011; Wappler et al., 2011). They also support axon growth both *in vitro* and *in vivo* (Frisen et al., 1995; Plantman et al., 2008). The expression of most of the prominent laminin chains are reduced in the adult brain, compared to development (Sanes, 1989). There are also deposits of laminin and laminin-like material seen in the aged brain, especially associated with AD (Jucker et al., 1996). Altered laminin isoforms in aging can impair endothelial cell function (Bell et al., 2010; Wagner et al., 2018). Alteration of laminin expression level and form in response to injury in older animals is likely to play an important role in maintaining BBB integrity and in participating in the reduction of axon growth.

#### Proteoglycans

Proteoglycans include dermatan sulfate proteoglycans (DSPGs), heparan sulfate proteoglycans (HSPGs), keratan sulfate proteoglycans (KSPGs) and CSPGs (George and Geller, 2018). CSPGs are the most studied in their role in pathfinding in development and restricting plasticity in adult CNS (Bartus et al., 2012). CSPGs are associated with glial scaring and reactive gliosis after SCI and inhibit axon growth. Thus, disruption of the CSPGs after SCI was thought to be a promising strategy to modify the glial scar, promote plasticity, axon growth and functional recovery. One strategy is the use of chondroitinase ABC (ChABC). There is flourishing literature on this topic (Muir et al., 2019). Proteoglycans composition changes during aging and accumulates in the brain (Reed et al., 2018; Richard et al., 2018). Increased proteoglycan sulfation with aging may reduce plasticity and memory (Foscarin et al., 2017). It will be of interest to assess the proteoglycans changes with age and SCI and to determine the effects of ChABC in middle-age and age animals.

#### Biomechanical Properties of the Injured Spinal Cord

The ECM composition alters the tissue stiffness. Stiffness has recently been advanced as an important factor for regeneration (Moeendarbary et al., 2017; Thompson et al., 2019). Changes in collagen, laminin, and increases in intermediate filaments GFAP and vimentin, modify the stiffness of the tissue. Axons growth decreases on a softer tissue, such as the glial scar in the SCI (Koser et al., 2016). Increase in tissue stiffness with age has been shown to reduce OPCs proliferation and differentiation (Segel et al., 2019). Therefore, changes of the ECM composition with age and injury are likely to alter the biomechanical properties and impede any attempt at promoting axon regeneration in aged animals.

#### Growth Factors

Numerous growth factors are known to promote axon regeneration, including IGFs, nerve growth factor (NGF), BDNF, glial cell-derived neurotrophic factor (GDNF), neurotrophins (NTs), fibroblast growth factors (FGFs), epidermal growth factors (EGF), and CNTF (Duraikannu et al., 2019).

##### Insulin-like growth factor

In the developing brain, IGF-1 promotes oligodendrocytes proliferation and differentiation and axonal growth (Ye et al., 2002; Dyer et al., 2016). IGF-1 has been shown to increase axon regeneration (Ozdinler and Macklis, 2006; Zhang Y. et al., 2019) although not in adult, most likely because of the lack of IGF-1 receptor (Hollis et al., 2009; Dupraz et al., 2013). Recently, IGF-1, only in combination with osteopontin, was shown to increase CST sprouting (Liu et al., 2017). IGF-1 expression levels decline with age. This reduction, associated with longevity, is proposed to be a neuroprotective after injury, as higher level of IGF-1 is associated with cognitive dysfunction (Shetty et al., 2005; Mao et al., 2018). Indeed, IGF-1/insulin inhibits axon regeneration in *C. elegans* in aging neurons (Byrne et al., 2014). Whether IGF-1 reduces regeneration in aged mammals needs to be determined. On the other hand, IGF-1 may have anti-inflammatory effect in the CNS (Sukhanov et al., 2007; Labandeira-Garcia et al., 2017) and may reduce the astrocytic response (Genis et al., 2014; Hernandez-Garzón et al., 2016). Therefore, a reduction of IGF-1 with age may participate to the age-related CNS inflammation and increase astrogliosis. Regardless, the involvement of the IGF1/IGF-1 receptor in the modulation of inflammation, astrogliosis and axon regeneration in the context of aging and neurotrauma is complicated.

##### Nerve growth factor

After SCI, NGF is increased (Brown et al., 2004). Infusion or overexpression of NGF increases axon growth after SCI (Oudega and Hagg, 1996; Romero et al., 2000) and reduces neuronal apoptosis, via activation of the PI3K/Akt and MAPK pathways (Zhang et al., 2014). However, NGF is decreased and its precursor, proNGF, is increased with aging (Al-Shawi et al., 2007; Budni et al., 2015). While proNGF promotes neurite growth in young rodents, it induces cell death but no growth in old animals (Al-Shawi et al., 2008). After SCI, proNGF mediates oligodendrocytes death (Beattie et al., 2002). Therefore, increase of proNGF with age after SCI is likely neurotoxic, and may not promote axon growth. However, it remains possible that infusion or overexpression NGF in older animal still promote axon growth after SCI.

##### Brain-derived neurotrophic factor

Brain-derived neurotrophic factor has repeatedly been shown to be neuroprotective and to enhance regeneration/sprouting after SCI through the MAPK, PI3K, and PLC γ signaling (Weishaupt et al., 2012; Garraway and Huie, 2016). However, it is not known if similar effects would be observed in older animals in the context of SCI. BDNF/TrkB is altered with age. TrkB receptor expression level declines with age in the pituitary (Rage et al., 2007) and aging is associated with BDNF and/or TrkB expression decrease at the neuromuscular junction (Greising et al., 2015; Greising et al., 2017). Reduction of BDNF with age is linked to cognitive, memory deficit and hippocampal shrinkage (Erickson et al., 2010; von Bohlen Und Halbach, 2010; Petzold et al., 2015). In sum, there is evidence that BDNF or its receptor, TrkB, are reduced in the context of injury in middle-age and aged animals. Thus, we speculate that BDNF-based treatments in older animal may still enhance axon growth after SCI but only to a limited degree, and that it may be necessary to combine them with other approaches, such as overexpression of TrkB in the neurons.

##### NT3

NT3 plays a role in neuron survival and axon regeneration (Keefe et al., 2017). NT3 overexpression also improves recovery after SCI by reducing dendritic atrophy and dendritic regrowth in young animals (Wang et al., 2018; Han et al., 2019). NT-3 binds preferentially to TrkC receptor (Keefe et al., 2017). However, a reduction of TrkC is observed with age in sensory neurons, which could reduce NT3 activity in older animals (Vaughan et al., 2017). Importantly, in a stroke model, delayed viral delivery of NT3 promotes recovery in both adult (6 months) and aged (18 months) rats, via increase in CST sprouting (Duricki et al., 2016). Therefore, the effects of this therapy on adult and elderly animals with SCI may also enhance CST sprouting, despite the potential reduction of TrkC in neurons.

##### Glial cell-derived neurotrophic factor

Member of the TGF-ß superfamily, GDNF has been shown to promote survival of neurons (Paratcha and Ledda, 2008). After SCI, administration of GDNF promotes functional recovery via different mechanisms, such as enhancing axon regeneration and sprouting, altering astrogliosis via reduction of GFAP and CSPGs production, reducing BSCB permeability and reduction of nitric oxide production. For review see Rosich et al. (2017). While a decrease in GDNF expression level with age and SCI would be expected, it has not been characterized. Similarly, the effect of GDNF delivery on functional recovery after SCI in age animals remains to be tested.

##### Fibroblast growth factors

Several members of the FGFs family, especially FGF1 and FGF2, have been shown to be involved in neuron survival and neurite extension (Teng et al., 1999). FGF1 also reduces inflammation (Lee et al., 2008) and astrogliosis (Lee et al., 2011; Kang et al., 2014). Different FGF treatment, alone or in combination, promote recovery after SCI (Rabchevsky et al., 2000; Zhou et al., 2018). Pathways activated by FGFs include ERK/MAPK, PLCγ and AKT. FGF2 and FGF receptors expression levels decline with age (Shetty et al., 2005; Hurley et al., 2016). However, changes of these factors and their receptors in the aged spinal cord have not been described. A decline of FGF receptors with age would indicate less potential for clinical translation potential and calls for further investigation.

##### Epidermal growth factors

Epidermal Growth Factors inhibits axon regeneration (Koprivica et al., 2005) and promotes reactive astrocytes (Liu et al., 2006; Codeluppi et al., 2009) through mTOR and AKT signaling. Inhibiting EGF receptor was found to promote functional recovery after SCI via modification of the glial scar, reducing inflammation and increasing OPC proliferation (Erschbamer et al., 2007; Li et al., 2011; Zhang S. et al., 2016). However, an opposite effect was later described, with an increase in lesion size and reduction in functional recovery (Berry et al., 2011; Sharp et al., 2012). EGF administration reduces BSCB disruption (Zheng et al., 2016). Decrease in EGF-Receptor with age has been documented in the skin fibroblasts (Shiraha et al., 2000), the aged hippocampus (Enwere et al., 2004) and other systems (Rongo, 2011; Siddiqui et al., 2012). How age and SCI affect expression levels of EGF and EGFR is not known. One could speculate that EGFR expression is reduced within the spinal cord of age animals, and that its inhibition, regardless of its positive or negative impact on recovery in young animals, will likely have minimal impact on functional recovery in aged animals.

While manipulation of the growth factors to promote axon growth after injury seem complex, the general consensus is that a combinatorial approach is needed. This has been elegantly demonstrated in the recent work from the Sofroniew lab (Anderson et al., 2018). In this study, 3 mechanisms were targeted: increase of the neuron-intrinsic capacity (with osteopontin, IGF-1 and CDNF), increase in growth support at the injury site (with FGF2 and EGF), and chemoattractant caudal to the injury (GDNF). Only the combination of these three factors lead to strong axon regeneration. These data warrant further testing in the context of aging.

### Myelin Proteins and Constituents

Studies have found multiple potent neurite growth inhibitory factors that are enriched in CNS myelin, such as Nogo-A, myelin-associated glycoprotein (MAG), oligodendrocyte/myelin glycoprotein (OMgp). After injury, myelin sheaths are damaged, and these factors are released into the extra-cellular environment. These molecules alter the regenerative potential after CNS and targeting them or their receptors can enhance axon growth after SCI, although, some conflicting results have been reported. Indeed, some groups reported a beneficial effect of genetic Nogo deletion on axon regeneration and sprouting and others only on sprouting (Cafferty et al., 2010; Lee et al., 2010). For review (Geoffroy and Zheng, 2014).

Few studies examined how age alters the expression of these proteins. Nogo-A levels are significantly decreased in aged animals (Trifunovski et al., 2006; Kumari and Thakur, 2014). A decrease in recovery is surprisingly observed in 12-month-old Nogo A/B deficient mice after TBI, compared to young adults, possibly related to hypomyelination (Marklund et al., 2009). Aged brains with stroke present a unique profile in growth-inhibitory molecule changes (Li and Carmichael, 2006). In aged brains OMgp is increased earlier than in young animals; MAG is increased in old injured brains but decreased in young; and Nogo-A is decreased in the aged while increased in young mice Nogo receptor 1 (NgR1) antagonist has been shown to increase OPC maturation and remyelination, and motor recovery after stroke in aged mice (Sozmen et al., 2016). While Nogo-A is not altered in 12-month-old mice, at least at 6 weeks post injury (Geoffroy et al., 2016), the exact age-related profile change of these molecules at different time point after SCI has not been characterized. Indeed, as discussed in Section “The Inflammatory Response Changes with Age: ‘Inflamm-Aging,” there are been reports of reduction in debris clearance by aged macrophages and monocytes (Neumann et al., 2008; Natrajan et al., 2015). Therefore, one would speculate that the inhibitory myelin proteins stay longer at the injury site of older animals, and reduce axon growth potential. Additionally, the expression of the MHC1 receptor, PirB, is increased with age in the hippocampus, localized primarily in neurons. Induction of the MHC1 pathway in neurons is potentially linked to increased neuro-immune signaling and altered synaptic homeostasis, contributing to age-related hippocampal dysfunction (VanGuilder Starkey et al., 2012). If a similar pattern occurs in spinal neurons, it may lead to greater inhibition in aged neurons than their younger counterparts with the same amount of myelin proteins. PirB is increased in the spinal cord after injury (Zhou et al., 2010), while how age alters spinal expression of PirB is still to be elucidated. Similarly, NgR1, and its ligands, are increased in the hippocampus with age (VanGuilder Starkey et al., 2013). These data suggest that, even absent alterations in myelin protein levels, changes in neuronal expression of receptors may increase the inhibitory effects of these proteins on axon regeneration.

## Conclusion

The regeneration of damaged axons has been extensively studied in the PNS, and it has long been accepted that there is an age-dependent decline in regeneration after injury. Axon injury in the CNS is a more complex matter, however, it has recently been recognized that the CNS also present an age-dependent decline in axon regeneration. Our current understanding of this decline is still limited and requires greater exploration. The aging population, increasing average age of SCI incidence and longer life-expectancies of people living with SCI, creates a strong need to increase our understanding of the age-dependent obstacles for axonal repair. Axon regeneration is dependent on a myriad of neuron intrinsic and extrinsic factors. The nature and balance of these factors can result in the promotion or inhibition of growth. After CNS injury, there is no significant axonal regeneration which has been associated with a variety of neuron-intrinsic elements as well as interactions with the injury environment. There is currently a lack of published literature reviews, and preclinical studies at the intersection of aging and SCI research. In this review we have concentrated on the impact of the interaction with the inflammatory response, the resident glial cells and the extracellular environment, and how these are altered both in an injury and in an aging paradigm. We propose that the interaction between injury and aging will compound the inherent difficulties and further inhibit the limited regenerative capability of the CNS. Finally, while this review focuses on the extrinsic factors that can influence the success of axon regeneration, the importance of the neuron-intrinsic factors and how these also interact with each other cannot be ignored.

## Author Contributions

TS and CG conceived and wrote the manuscript.

## Conflict of Interest

The authors declare that the research was conducted in the absence of any commercial or financial relationships that could be construed as a potential conflict of interest.

## Abbreviations

AD: Alzheimer’s disease
ALS: amyotrophic lateral sclerosis
BBB: blood-brain barrier
BDNF: brain-derived neurotrophic factor
BMP: bone morphogenetic protein
BSCB: blood-spinal cord barrier
ChABC: chondroitinase ABC
CNS: central nervous system
CNTF: ciliary neurotrophic factor
CSPG: chondroitin sulfate proteoglycans
CST: cortico-spinal tract
DSPG: dermatan sulfate proteoglycans
EGF: epidermal growth factor
ERK: extracellular signal-regulated kinases
FGF: fibroblast growth factors
GABA: gamma aminobutyric acid
GDNF: glial cell-derived neurotrophic factor
GFAP: glial fibrillary acidic protein
HD: Huntington’s disease
HSPG: heparan sulfate proteoglycans
IGF: insulin-like growth factor
IL: interleukin
JAK: Janus kinase
JNK: C-JunN-Terminal Kinase
KLFs: Krüppel-like factors
KSPG: keratan sulfate proteoglycans
LPS: lipopolysaccharide
MAG: myelin-associated glycoprotein
MAPK: mitogen activated protein kinase
MEK: mitogen-activated protein kinase kinase
mTOR: mammalian target of rapamycin
NF κ B: nuclear factor Kappa-light-chain-enhancer of activated B cells
NGF: nerve growth factor
NOX: NADPH-oxidase
NTs: neurotrophins
OMgp: oligodendrocyte myelin glycoprotein
OPCs: oligodendrocyte progenitor cells
PNS: peripheral nervous system
PTEN: phosphatase and tensin homolog
RNS: reactive nitrogen species
ROS: reactive oxygen species
SCI: spinal cord injury
SOCS3: suppressor of cytokine signaling 3
STAT3: signal transducer and activator of transcription 3
TBI: traumatic brain injury
TGF: transforming growth factor
TNF: tumor necrosis factor
VEGF: vascular endothelial growth factor.
